# Neuronal uptake of anti-Hu antibody, but not anti-Ri antibody, leads to cell death in brain slice cultures

**DOI:** 10.1186/s12974-014-0160-0

**Published:** 2014-09-17

**Authors:** John E Greenlee, Susan A Clawson, Kenneth E Hill, Blair Wood, Stacey L Clardy, Ikuo Tsunoda, Troy D Jaskowski, Noel G Carlson

**Affiliations:** Neurology Service, George E. Wahlen Veterans Affairs Medical Center, 500 Foothill Drive, Salt Lake City, UT 84148 USA; Department of Neurology, University of Utah School of Medicine, 50 North Medical Drive, Salt Lake City, UT 84132 USA; Brain Institute, University of Utah, 383 Colorow Drive, Salt Lake City, UT 84108 USA; Department of Microbiology and Immunology, Louisiana State University Health Science Center, 1501 Kings Highway, Shreveport, LA 71130 USA; Institute for Clinical and Experimental Pathology, ARUP, 500 Chipeta Way, Salt Lake City, UT 84108 USA; GRECC, George E. Wahlen Veterans Affairs Medical Center, 500 Foothill Drive, Salt Lake City, UT 84148 USA; Department of Neurobiology and Anatomy, University of Utah School of Medicine, 50 North Medical Drive, Salt Lake City, UT 84132 USA; Center on Aging, University of Utah, 10 South 2000 East, Salt Lake City, UT 84112-5880 USA

**Keywords:** Paraneoplastic syndromes, Anti-Hu antibody, Anti-Ri antibody, Neurons, Brain slice cultures, Organotypic brain cultures, Cell death, Apoptosis, Hu antigens ANNA-1, ANNA-2

## Abstract

**Background:**

Anti-Hu and anti-Ri antibodies are paraneoplastic immunoglobulin (Ig)G autoantibodies which recognize cytoplasmic and nuclear antigens present in all neurons. Although both antibodies produce similar immunohistological labeling, they recognize different neuronal proteins. Both antibodies are associated with syndromes of central nervous system dysfunction. However, the neurological deficits associated with anti-Hu antibody are associated with neuronal death and are usually irreversible, whereas neurological deficits in patients with anti-Ri antibody may diminish following tumor removal or immunosuppression.

**Methods:**

To study the effect of anti-Hu and anti-Ri antibodies on neurons, we incubated rat hippocampal and cerebellar slice cultures with anti-Hu or anti-Ri sera from multiple patients. Cultures were evaluated in real time for neuronal antibody uptake and during prolonged incubation for neuronal death. To test the specificity of anti-Hu antibody cytotoxic effect, anti-Hu serum IgG was incubated with rat brain slice cultures prior to and after adsorption with its target Hu antigen, HuD.

**Results:**

We demonstrated that: 1) both anti-Hu and anti-Ri antibodies were rapidly taken up by neurons throughout both cerebellum and hippocampus; 2) antibody uptake occurred in living neurons and was not an artifact of antibody diffusion into dead cells; 3) intracellular binding of anti-Hu antibody produced neuronal cell death, whereas uptake of anti-Ri antibody did not affect cell viability during the period of study; and 4) adsorption of anti-Hu antisera against HuD greatly reduced intraneuronal IgG accumulation and abolished cytotoxicity, confirming specificity of antibody-mediated neuronal death.

**Conclusions:**

Both anti-Hu and anti-Ri antibodies were readily taken up by viable neurons in slice cultures, but the two antibodies differed markedly in terms of their effects on neuronal viability. The ability of anti-Hu antibodies to cause neuronal death could account for the irreversible nature of paraneoplastic neurological deficits in patients with this antibody response. Our results raise questions as to whether anti-Ri antibody might initially induce reversible neuronal dysfunction, rather than causing cell death. The ability of IgG antibodies to access and react with intracellular neuronal proteins could have implications for other autoimmune diseases involving the central nervous system.

**Electronic supplementary material:**

The online version of this article (doi:10.1186/s12974-014-0160-0) contains supplementary material, which is available to authorized users.

## Background

Anti-Hu (antineuronal nuclear antibody 1; ANNA1) and anti-Ri (antineuronal nuclear antibody 2; ANNA2) antibodies are paraneoplastic autoantibodies which react with nuclear and cytoplasmic proteins of neurons throughout the central nervous system. Anti-Hu antibody is closely associated with small cell carcinoma of the lung and has been reported in patients with a wide spectrum of paraneoplastic neurological disorders, including limbic encephalitis, cerebellar degeneration, brainstem encephalitis, dorsal sensory neuronopathy, motor neuron disease, and gastroparesis [1,2]. Anti-Ri antibody, found predominantly in patients with breast adenocarcinomas and small cell carcinoma of the lung, has been associated with syndromes of opsoclonus/ataxia, cerebellar degeneration, limbic or brainstem encephalitis, ophthalmoplegia, laryngeal dystonia, and gastroparesis [3–12]. Although anti-Hu and anti-Ri antibodies produce essentially identical patterns of immunohistological labeling in human or rodent brain sections [13], the antigens recognized by the two antibodies are distinct and are encoded by different genes [14–16]. Anti-Hu antibody recognizes proteins of 35-42 kDa in Western blots of neuronal lysates, whereas anti-Ri antibody recognizes antigens of 55 kDa [1,5]. Paraneoplastic neurological syndromes associated with anti-Hu antibodies are accompanied by neuronal destruction and usually show no clinical improvement following immunosuppressive treatment or tumor removal [17–22]. Although some degree of neuronal destruction has been described in cases of anti-Ri encephalomyelitis, findings have been more variable [3,4,23,24], and clinical improvement has been reported in some patients with anti-Ri antibody following immunosuppressive therapy or successful treatment of the underlying malignancy [5,6,18,23–28].

Despite repeated detection of anti-Hu and anti-Ri antibodies in sera and cerebrospinal fluid of affected patients, a direct role for either antibody in the pathogenesis of paraneoplastic neurological injury has been considered unlikely [29,30]. Viable neurons have been widely believed to exclude immunoglobulin (Ig)G [29,30]. Because both anti-Hu and anti-Ri antibodies recognize intracellular antigens, it has been thought that neither antibody would be able to react with its target antigens *in vivo* to induce disease [29,30]. We and others have previously reported destruction of neurons by anti-Hu antibody in dispersed cell cultures [31,32]; however, the relevance of these findings to events occurring *in vivo* has been uncertain, and attempts by others to produce neurological injury in experimental animals by immunization with recombinant Hu antigens have been unsuccessful [33].

To study the interaction of antineuronal antibodies with neurons, we have established a brain slice (organotypic) culture system which preserves anatomical relationships present *in vivo* and allows exposure of neurons to antibodies without interposition of the blood-brain barrier [34,35]. We have previously demonstrated that living Purkinje cells in cerebellar slice cultures incorporated and subsequently cleared normal IgG. Although the intracellular presence of normal IgG *per se* did not affect Purkinje cell viability, incubation of cultures with an IgG-daunorubicin immunotoxin resulted in Purkinje cell uptake of the immunotoxin and targeted Purkinje cell death [35]. We have subsequently demonstrated that the paraneoplastic autoantibody anti-Yo, associated with cerebellar degeneration, was taken up by Purkinje cells, and that intracellular accumulation of anti-Yo antibody resulted in non-apoptotic Purkinje cell death [34].

In the present study, using rat cerebellar and hippocampal slice cultures, we examined whether anti-Hu and anti-Ri antibodies might also be taken up by Purkinje cells or other neuronal populations and whether uptake of either antibody was associated with neuronal death. We also assessed whether specific binding of anti-Hu antibodies to intracellular Hu antigen was required for antibody-mediated cytotoxicity. We found that both antibodies were taken up by neurons and that binding of anti-Hu antibody to its intracellular target antigens induced neuronal death over time, whereas anti-Ri antibody did not induce cell death during the period of study.

## Methods

### Patient materials

Sera from nine patients with paraneoplastic neurological disorders were studied. Seven of these patients had anti-Hu antibodies and two patients had anti-Ri antibodies. The presence of anti-Hu or anti-Ri antibodies and absence of other known paraneoplastic autoantibodies was confirmed in all patients by: 1) immunohistological staining of neurons typical for anti-Hu or anti-Ri antibody in frozen and fixed sections of human and rat cerebellum; 2) antibody binding restricted to the 35-42 kDa proteins characteristic of Hu antigens or the 55 kDa antigen recognized by anti-Ri antibody in Western blots of neuronal lysates; and/or 3) commercial identification of Hu or Ri antibodies (ARUP, University of Utah, USA) [36]. Serum antibody titers by endpoint dilution ranged from 1:320 to 1:100,000. Control serum samples were from individuals without cancer or neurological disease. All materials were obtained and studied under Institutional Review Board guidelines (University of Utah and Veterans Affairs Salt Lake City Health Care System, USA), either under written informed consent or as de-identified patient samples. Purified IgG was prepared from patient sera by column chromatography as previously described [31].

### Preparation of slice (organotypic) cultures

All aspects of animal handling and care were conducted with local Institutional Animal Care and Use Committee approval in an Association for Assessment and Accreditation of Laboratory Animal Care-approved facility and were conducted according to applicable national and international guidelines. Slice cultures were prepared from the two brain regions most commonly affected in patients with anti-Hu or anti-Ri antibody-associated paraneoplastic syndromes: cerebellum, given the association of the two antibodies with cerebellar degeneration and/or syndromes of opsoclonus-ataxia; and hippocampus, given the association of both antibodies with limbic encephalitis. Limited studies were also done employing slice cultures including the cerebral cortex. Sprague-Dawley rats, ages p10-12 or p23-27 days (Charles River, Germantown, MD, USA), were euthanized with carbon dioxide and cerebellar and hippocampal slice cultures prepared at 250-350 μm thickness using the method of Rothstein and colleagues, as previously described [34,35,37]. Horse sera (Life Technologies Grand Island, NY, USA) used in tissue culture media were heated to inactivate complement. Cultures were incubated at 37°C in a 5% CO_2_/95% humidified air environment with twice weekly changes of medium. Sera and purified IgGs were used at dilutions of 1:400 to approximate amounts of antineuronal antibodies typically found in patient cerebrospinal fluid.

### Detection of neuronal cell death and apoptosis

To detect cell death, we incubated cultures for the final 2 hours prior to harvesting with SYTOX cell viability dyes (SYTOX green or SYTOX orange; Invitrogen, Springfield, OR, USA). SYTOX dyes are fluorescent compounds which stain intracellular nucleic acids; these dyes are excluded from living cells but readily enter cells following cell membrane injury and death [38]. We have previously demonstrated the utility of these dyes in detecting dead or dying neurons in brain slice cultures [34,35]. Detection of antibody-containing neurons undergoing apoptosis employed the pan-caspase marker FLICA (fluorochrome-labeled inhibitors of caspases; carboxyfluorescein-labeled fluoromethyl ketone peptide inhibitor of caspases; Immunochemistry Technologies, LLC, Bloomingdale, MN, USA) [34]. To detect both cell death and apoptosis, cultures incubated with anti-Hu antibodies, anti-Ri antibodies, or control IgGs were treated for the last 4 hours prior to harvesting with a 1:5 dilution of FLICA and, for the last 2 hours prior to harvesting, with SYTOX dyes [39,40]. Confirmation of apoptosis in cultures exhibiting FLICA staining was carried out in selected replicate cultures using a terminal deoxynucleotidyl transferase dUTP nick end labeling (TUNEL) *in situ* cell death detection kit (TMR red, Roche Applied Science, Indianapolis, IN, USA). In these experiments, cultures were incubated with 1:400 dilutions of patient or control sera for 72 hours. FLICA was added to cultures 4 hours prior to fixation and SYTOX dyes were added 2 hours prior to harvesting. Cultures were then fixed in 2% paraformaldehyde, permeabilized, and incubated with TUNEL assay mix at 37°C for 2 hours. Uptake of antibody was confirmed by immunofluorescence staining using Cy5-conjugated donkey anti-human IgG, and cell death was confirmed by SYTOX green exclusion as well as by FLICA or TUNEL staining. Positive controls for apoptosis included permeabilized cultures treated with DNase I (Sigma-Aldrich, St Louis, MO, USA) to induce nicks in DNA to allow TUNEL staining. Negative controls included cultures maintained without IgG, with normal IgG, or with omission of conjugated secondary antibody during postfixation staining. Negative TUNEL controls were cerebellar cultures incubated with the TUNEL assay mix without addition of DNase.

### Investigation of anti-Hu or anti-Ri antibody uptake and accumulation by viable neurons in real time

To exclude the possibility that detection of intraneuronal IgG might be due to leakage of intravascular Igs into brain parenchyma and neurons after death [41,42], we studied the interaction of anti-Hu and anti-Ri antibodies with neurons in real time as previously described [34]. IgGs containing high titers of anti-Hu or anti-Ri antibody were purified and conjugated to Cy5 using a DyLight 650 Antibody Labeling Kit (Thermo Scientific/Pierce Biotechnology, Rockford, IL, USA) [34]. Cerebellar and hippocampal cultures incubated with anti-Hu or anti-Ri IgGs conjugated to Cy5 were observed at intervals through 24 hours using a microscope stage incubating chamber (SmartSlide microincubation chamber, WaferGen Biosystems, Fremont, CA, USA) and a Nikon Eclipse TE300 inverted microscope (Nikon Biosciences, Melville, NY, USA.

To confirm that neurons in these cultures were viable and that uptake of Cy5-conjugated anti-Hu or anti-Ri antibody seen in fixed cultures corresponded to stages of antibody uptake observed in real time, SYTOX green was added to each culture, and the cultures were fixed with 2% paraformaldehyde after 6 and 24 hours of incubation and subjected to confocal analysis. To minimize artifactual labeling of cells resulting from diffusion of extracellular IgG, fixed cultures incubated with Cy5-conjugated IgG in these real time studies were not subjected to permeabilization with Triton X-100. Distribution of IgG within neurons was compared with that seen in the same cultures during observation in real time.

### Investigation of anti-Hu or anti-Ri antibody uptake and cytotoxicity in cultures during prolonged incubation

Cerebellar and hippocampal cultures were incubated with 1:400 dilutions of anti-Hu or anti-Ri sera or corresponding dilutions of normal sera or purified normal IgG. Cultures were harvested at 24-hour intervals from 24 to 144 hours, with limited additional cultures being maintained for 168 to 219 hours. All studies, unless otherwise indicated, were performed in triplicate. Cultures were treated with FLICA and SYTOX and then fixed in 2% paraformaldehyde, permeabilized with 0.2% Triton X-100, treated with ImageiTFx signal enhancer (Life Technologies, Molecular Probes, Eugene, OR, USA), and incubated overnight at 4°C with 1:400 dilutions of Cy5 (Alexa Fluor 647)-conjugated donkey anti-human IgG (Jackson ImmunoResearch, West Grove, PA, USA) as previously described [35].

### Quantification of neuronal death

Sera from seven patients with anti-Hu antibody response and two patients with anti-Ri antibody response were studied. Cultures were incubated for 72 hours with control, anti-Hu, or anti-Ri sera. FLICA was added 4 hours prior to fixation and SYTOX orange was added 2 hours prior to fixation as previously described [34]. Amounts of apoptosis and cell death as determined by immunofluorescence microscopy were quantified by an observer unaware of treatment of the cultures and consisted of counting the number of cells labeled by Cy5-conjugated anti-human IgG that were stained by SYTOX orange or FLICA or which remained unstained by either agent. Live cells were recorded as containing IgG but without staining by either SYTOX orange or FLICA. Dead cells were scored as cells co-labeled for IgG and SYTOX orange, FLICA, or both. Approximately 40 to 90 cells were counted for each field and the average percentage cell death was obtained from a minimum of eight fields captured at 40× magnification for each time point [35]. Neurons in control cultures did not contain amounts of IgG detectable by immunofluorescence. For purposes of quantification, neurons in these cultures were identified by postfixation immunostaining of cultures with anti-Hu IgG followed by Cy5-conjugated donkey anti-human IgG. Statistical significance between groups was determined by non-parametric Kruskal-Wallace test with Dunn’s multiple comparison test using GraphPad Instat statistical software (GraphPad Software, Inc., La Jolla, CA, USA) [34,35].

### Analysis of immunoglobulin G accumulation and neuronal death in cultures incubated with human sera depleted of antibody to the HuD antigen

HuD is a recombinant cDNA cloned from a lambda zap library using anti-Hu antibodies and is widely used as a specific antigenic target in testing for anti-Hu antibody response [15]. Despite its use in diagnostic testing, however, the role of antibodies to the HuD protein in the pathogenesis of neuronal death has not been established [15]. To determine whether antibodies reactive with the HuD protein were required for neuronal cytotoxicity, we compared neuronal accumulation of anti-Hu patient IgG and neuronal death before and after the antiserum had been adsorbed to recombinant HuD protein. In these studies, we used anti-Hu IgG which we had shown to produce extensive cell death in both cerebellar and hippocampal cultures. The coding region (1101 nucleotides, 366 amino acids) for the HuD protein (homo sapiens ELAV, embryonic lethal, abnormal vision, Drosophila-like 4, accession number BC036071.1) [15] was sub-cloned into the plasmid pReceiver-B31 that contains a T7 promoter and a multiple cloning site which allows in-frame fusion of six histidine residues (His tag which facilitates binding to a nickel column) to the C-terminus of the protein. The resulting plasmid (EX-Z-0448-B31) was constructed by GeneCopoeia (Rockville, MD, USA) and transformed into the *E. Coli* strain BL21 (DE3)/pLysS. Post-induction cell pellets were frozen in buffer (NTB- Clontech) containing protease inhibitors, and the cell lysate containing the 40 kDa recombinant HuD-His tag protein was adsorbed onto a nickel affinity column (Clontech column #635657). As a sham control for nonspecific binding, the same treatment was performed with bacteria containing a control vector which lacked the His-tag HuD antigen. The anti-Hu patient IgG (diluted 1:100 in organotypic culture media) was passed over the His-tagged HuD antigen column or over the sham control nickel column treated with *E. coli* lysate containing the control vector but lacking the His-tag HuD antigen. Eluates from each column were tested by dot blot assays (spotted with recombinant His-tag HuD antigen) and showed that immunoreactivity to the HuD protein was removed when passed over the His-tag HuD antigen nickel column but was unaffected by passing over the column with control vector lacking the His-tag HuD antigen (data not shown).

### Confocal microscopy

Confocal analysis employed a Nikon Eclipse E800 upright microscope (Nikon Biosciences) and the Personal Confocal Microscopy PCM-2000 utilizing Argon-ion and green and red HeNe lasers. Simple Personal Confocal Image software program (Compix, Cranberry Township, PA, USA) was used to acquire digital images and image analysis. A red HeNe laser with a 633 nm excitation filter and 675 LP filter was used to visualize Cy5. An Argon-ion laser with a 514 nm excitation filter was used with a 510 LP filter to image SYTOX green. A green HeNe laser with a 543 nm excitation filter and a 565 LP filter was used to visualize SYTOX orange. The argon laser with a 488 nm excitation filter and a 510 LP filter was used to visualize FLICA. All filters were matched to the peak emission spectra of the fluorochromes employed. General procedures utilized individual fluorochromes with X, Y, and Z scans of 14 to 20 focal planes. Identical focal plane settings for each fluorochrome were used for single visual field analysis to ensure that each corresponding fluorochrome was imaged in the same focal plane. In all studies, stringent uniform experimental parameters and computer software settings were maintained for the respective image analyses. Because the vibratome preparation techniques used to prepare cerebellar slice cultures invariably resulted in death of neurons on the cut surfaces of culture slices, image analyses in all experiments were confined to the interior portions of the cultures.

## Results

### Uptake of anti-Hu and anti-Ri antibodies can be demonstrated in real time in viable cerebellar and hippocampal neurons

**Figure 1:**
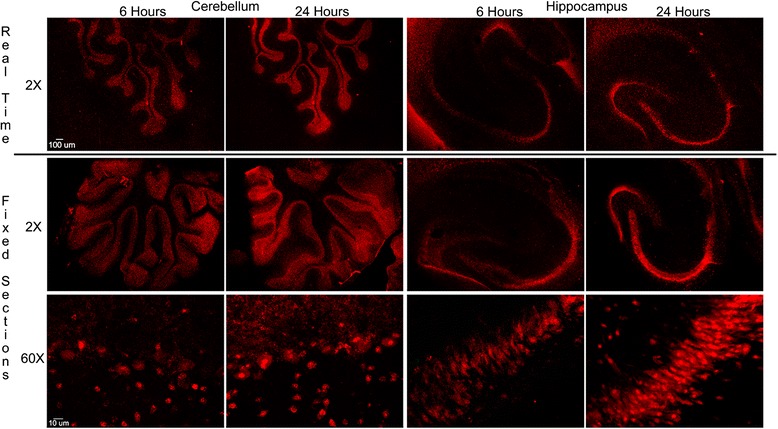
**Real time uptake of Cy5-conjugated anti-Hu immunoglobulin G in living cultures compared with uptake in parallel cultures examined following fixation.** Cultures were incubated with Cy5-conjugated anti-Hu immunoglobulin (Ig)G and were examined *in situ* for antibody uptake at 6 and 24 hours. Anti-Hu IgG was visible within multiple neuronal populations at 6 hours, with an increase in numbers of antibody-containing neurons and intensity of immunolabeling for intraneuronal IgG by 24 hours. Parallel cultures at both time points were treated with SYTOX dyes and fixed with 2% paraformaldehyde. Uptake of Cy5-conjugated anti-Hu IgG in these cultures was indistinguishable from that observed in slice cultures studied in real time, confirming that IgG uptake observed in cultures fixed following incubation with antibody accurately reflected events occurring prior to fixation. Neurons containing Cy5-conjugated anti-Hu IgG at these time points excluded SYTOX dyes, indicating that antibody uptake had occurred in living cells. Images are representative of real-time studies employing anti-Hu sera from two different patients, carried out in triplicate. Magnification bar indicates 20 μm.

**Figure 2:**
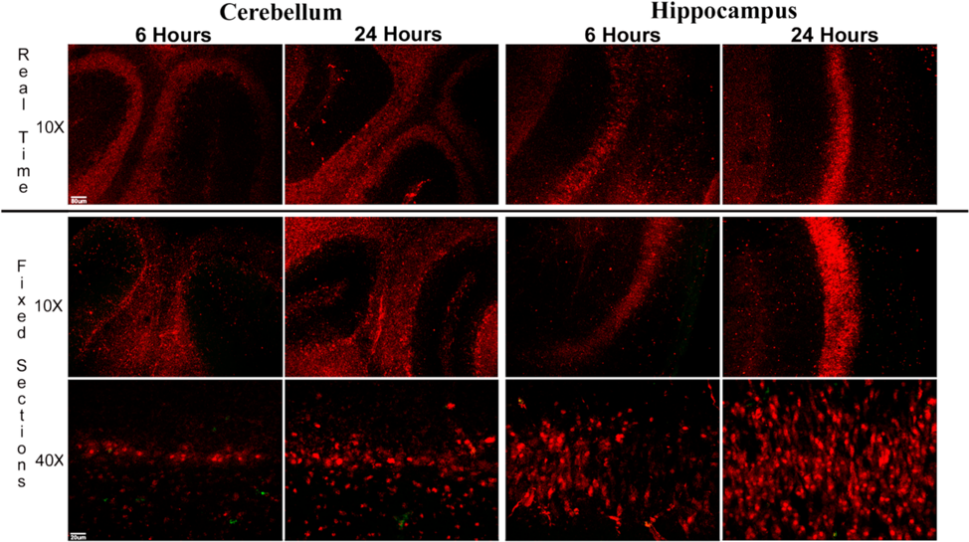
**Real-time uptake of Cy5-conjugated anti-Ri immunoglobulin G in living cultures compared with uptake in parallel cultures following fixation at the same time points.** In cultures studied in real time, Cy5 conjugated anti-Ri immunoglobulin (Ig)G was visible within multiple neuronal populations at 6 hours, with an increase in intensity of intraneuronal IgG by 24 hours, similar to observations with anti-Hu IgG. Uptake of Cy5-conjugated anti-Ri IgG in parallel cultures fixed following incubation with antibody were indistinguishable from that observed in slice cultures studied in real time, confirming that IgG uptake seen in fixed cultures accurately reflected events occurring prior to fixation. Neurons containing Cy5-conjugated anti-Ri IgG at both time points excluded SYTOX dyes, indicating that antibody uptake had occurred in living cells. Images are representative of real-time studies employing anti-Ri sera from two different patients, carried out in triplicate. Magnification bar indicates 20 μm.

A potential concern in studies of antibody uptake by neurons is that residual extracellular anti-Hu or anti-Ri antibodies present in cultures following incubation might enter neurons during fixation and permeabilization and result in detectable levels of antibody binding to intracellular Hu or Ri antigens [41]. To exclude this possible artifact, cerebellar and hippocampal cultures maintained in a microscope stage incubation chamber were incubated with 1:400 dilutions of either anti-Hu IgG or anti-Ri IgG conjugated to Cy5. This allowed us to examine by confocal microscopy whether IgG containing anti-Hu or anti-Ri antibody was taken up in real time prior to any fixation. In cultures incubated with Cy5-conjugated IgG from anti-Hu positive patients, bright fluorescence was detected in neurons at 6 hours, with significant increase in uptake by 24 hours (Figure 1). Uptake and accumulation of anti-Hu IgG in cerebellar cultures was observed in Purkinje cells, granule cells, and molecular layers. In hippocampal cultures, anti-Hu IgG was found in neurons in all anatomical regions including the pyramidal layer and the dentate gyrus. Similar widespread neuronal uptake of IgG was seen in cultures incubated with anti-Ri IgG (Figure 2). Distribution of Cy5-conjugated anti-Hu or anti-Ri IgG within neurons excluding SYTOX cell death dyes in fixed cultures studied at each time point was identical to that seen in the same cultures studied in real time (Figures 1 and 2). These observations demonstrate that neuronal uptake of anti-Hu or anti-Ri IgG in both cerebellar and hippocampal cultures had occurred prior to fixation while cells were viable and that observations made using fixed cultures reflected those seen in living cells prior to harvesting (Figures 1 and 2).

### Localization of anti-Hu and anti-Ri antibody binding in cerebellar and hippocampal neurons

**Figure 3:**
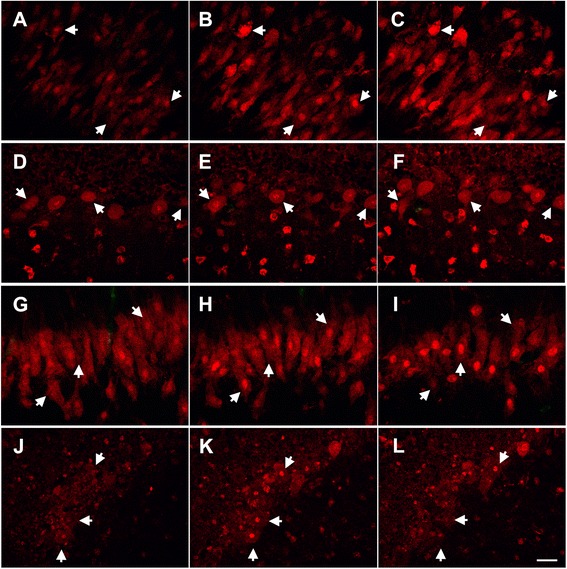
**Demonstration of cytoplasmic and nuclear accumulation of antibody in cultures incubated with anti-Hu or anti-Ri antibody.** The intracellular distribution of anti-Hu or anti-Ri antibody was assessed after cultures were incubated with either anti-Hu or anti-Ri antibody, for 6, 24 or 72 hours, using serial confocal images through individual neurons. Anti-Hu and anti-Ri immunoglobulin (Ig)G could be detected in both cytoplasm and nuclei of neurons as early as after 6 hours of incubation, with progressive accumulation of antibody over time. Neurons incorporating IgG excluded SYTOX dyes, indicating that antibody uptake had occurred in living cells. **(A-C)** Serial images of neurons within hippocampal slice cultures incubated with anti-Hu antibody for 24 hours. **(D-F)** Serial images of neurons within cerebellar slice cultures incubated with anti-Hu antibody for 24 hours. **(G-I)** Images of neurons in slice cultures of hippocampus following 72 hours of incubation with anti-Ri antibody. **(J-L)** Images of neurons in slice cultures of cerebellum following 72 hours of incubation with anti-Ri antibody. Arrows identify the same individual neurons at different levels in each of the three images. Magnification bar indicates 20 μm.

To assess the intracellular distribution of anti-Hu or anti-Ri antibodies after incorporation by neurons, serial confocal images through individual neurons were obtained after 6, 24 and 72 hours of incubation. In these cultures, anti-Hu and anti-Ri IgG could be detected in both cytoplasm and nuclei of neurons within as early as 6 hours after incubation, with progressive accumulation of antibody over time (Figure 3; Additional file 1). Neurons incorporating IgG excluded SYTOX dyes, indicating that antibody uptake had occurred in living cells. The distribution of intraneuronal IgG in cultures incubated with anti-Hu or anti-Ri antibody was essentially identical to the reported patterns of labeling produced by these two antibodies in conventional immunohistological studies of fixed tissue [1,4].

### Anti-Hu antibodies induce cell death and apoptosis in cerebellar and hippocampal cultures

**Figure 4:**
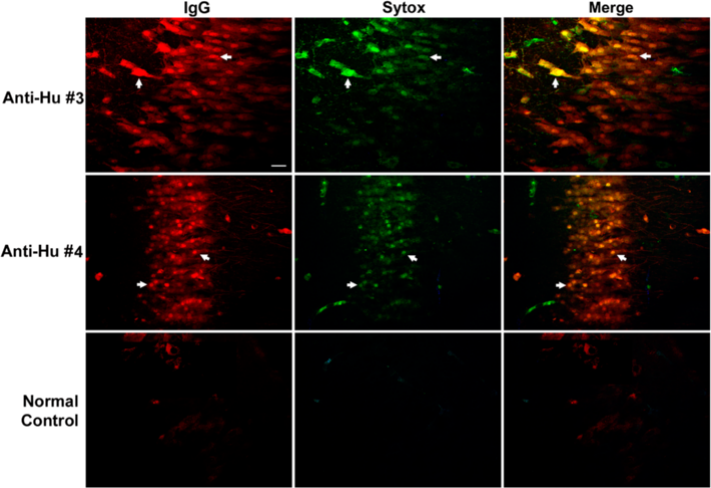
**Uptake of anti-Hu antibodies by hippocampal neurons was followed by neuronal death.** Hippocampal slice cultures from rat pups at 23 to 24 days of age were incubated for 72 hours with a 1:400 dilution of either anti-Hu or normal control sera. SYTOX green was added to cultures 2 hours prior to harvesting as a marker of cell death. Cultures were fixed and immunostained with Cy5-conjugated donkey anti-human immunoglobulin (Ig)G (red). Multiple neurons exhibited yellow fluorescence, due to the presence of both anti-Hu IgG (red) and SYTOX green, indicative of cell membrane disruption and death in neurons containing anti-Hu IgG. Images shown are representative of experiments investigating all seven anti-Hu samples studied. IgG uptake was not observed in control cultures incubated with normal human IgG, and only rare neurons stained with SYTOX dyes. Magnification bar = 20 μm.

To determine whether anti-Hu IgG caused neuronal cell death in cerebellar or hippocampal cultures, we incubated cultures for periods of up to 168 hours with sera from seven patients with anti-Hu antibody response. Uptake of IgG by neurons throughout cerebellar and hippocampal cultures was observed after 6 hours in all cultures incubated with anti-Hu sera, identical to our findings in real-time experiments (data not shown). Neurons in cultures examined after incubation with anti-Hu sera for 24 and 48 hours did not stain with SYTOX cell death dyes above levels seen in control cultures incubated with normal sera. By 72 hours, however, individual neurons in cultures incubated with anti-Hu sera contained SYTOX dyes, indicative of cell membrane disruption and death (Figure 4; Additional file 2). Numbers of dead neurons continued to increase over incubation periods of up to 168 hours.

**Figure 5:**
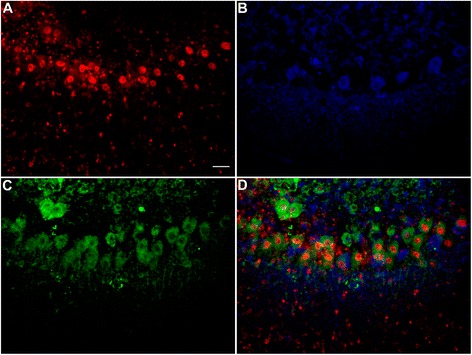
**Induction of neuronal apoptosis by anti-Hu antibody. (A)** Rat cerebellar slice culture incubated with anti-Hu serum for 72 hours and labeled with Cy5-conjugated donkey anti-human immunoglobulin (Ig)G (red), showing neuronal uptake of IgG. **(B)** Same culture showing fluorochrome-labeled inhibitors of caspases (FLICA) staining of neurons (blue), indicative of apoptosis. **(C)** Same culture stained with SYTOX dyes (green). There was extensive staining of neurons indicative of cell membrane destruction and death. **(D)** Merged image demonstrating that IgG-containing cells stained with both FLICA and SYTOX. Images are representative of studies employing anti-Hu sera from all seven patients, carried out in triplicate. Magnification bar = 20 μm.

**Figure 6:**
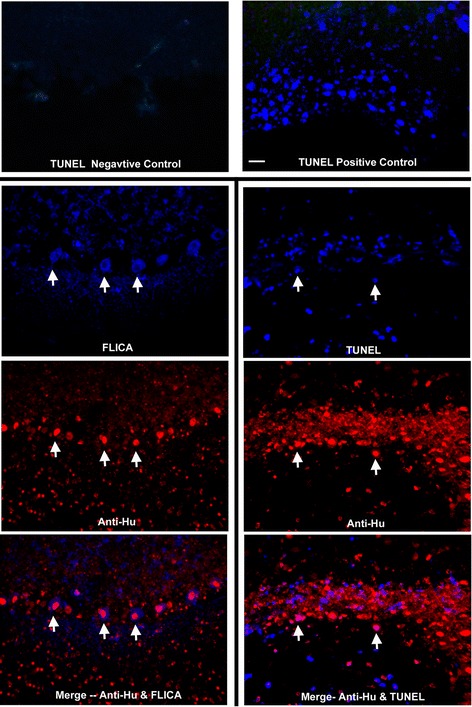
**Detection of anti-Hu antibody induced apoptotic neuronal death using FLICA staining as compared with TUNEL staining.** The top two panels show terminal deoxynucleotidyl transferase dUTP nick end labeling (TUNEL) negative and positive controls (see Methods). In the bottom panels arrows indicate the same neurons showing fluorochrome-labeled inhibitors of caspases (FLICA) or TUNEL staining (blue), containing immunoglobulin (Ig)G (red), or in merged images showing both IgG and FLICA or TUNEL staining (pink). Magnification bar = 20 μm.

To determine whether neuronal death associated with anti-Hu antibodies involved apoptosis, cerebellar and hippocampal cultures incubated with anti-Hu antibody for 72 hours were subsequently incubated with FLICA to detect apoptosis and SYTOX orange to detect cell death. Apoptotic cells, as determined by FLICA staining, were detected in both cerebellar and hippocampal cultures incubated with anti-Hu antibody, with some neurons staining with both SYTOX dyes and FLICA (Figure 5). Apoptosis as detected by FLICA staining was confirmed using immunofluorescent TUNEL methods (Figure 6) [34].

### Quantification of neuronal death in cultures incubated with anti-Hu antibody

**Figure 7:**
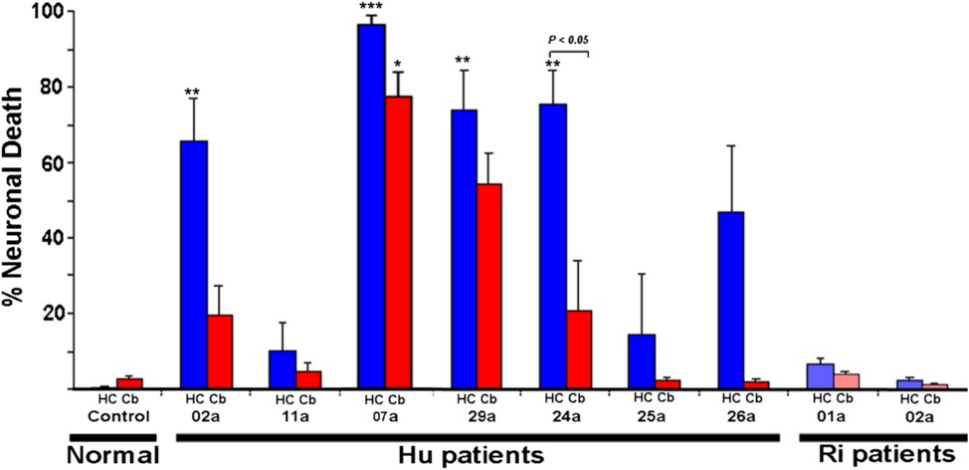
**Quantification of neuronal death associated with uptake of anti-Hu and anti-Ri antibodies in rat hippocampal and cerebellar slice cultures.** The percentage of dead neurons containing immunoglobulin (Ig)G is shown for control, anti-Hu, and anti-Ri patient sera for hippocampal (HC; blue bars) and cerebellar (Cb; red bars) cultures. Although a majority of anti-Hu samples produced neuronal death above controls, considerable variation in cytotoxicity was observed (see Results). Larger numbers of neurons labeled by death markers were seen in hippocampal cultures than in cerebellar cultures; however, this reached statistical significance in only one patient (patient 24a). Cultures exhibiting statistically significant death as compared to cerebellar or hippocampal cultures incubated with normal IgG, using Kruskal-Wallis nonparametric analysis of variance and Dunns multiple comparison test, are indicated by asterisks (*P < 0.05; **P < 0.01; ***P < 0.001).

Neuronal death was quantified in cultures treated for 72 hours with anti-Hu, anti-Ri, or control sera (see Methods) (Figure 7). Seven anti-Hu sera and two anti-Ri sera were studied. The extent of apoptotic and non-apoptotic cell death in neurons containing IgG, as determined by FLICA staining and by entry of SYTOX dyes, was compared with that seen in normal serum controls as previously described [34]. Hippocampal and cerebellar cultures incubated with control sera exhibited less than 5% background neuronal death (Figure 7). In contrast, although variation in amounts of cell death was seen among anti-Hu sera studied, four of the sera produced death in up to 90% of hippocampal neurons and 80% of cerebellar neurons over 72 hours of incubation. Increased neuronal death was also observed in cerebellar and hippocampal cultures incubated with the other three anti-Hu sera, but statistical significance was not achieved compared with controls. Numbers of dead neurons in hippocampal cultures exceeded those in cerebellar cultures for each serum studied. However, this difference between hippocampal and cerebellar cultures reached statistical significance with only one sample. The apparent differences in cytotoxicity could not be accounted for by differences in antibody uptake or binding as determined by immunohistological methods, and it is possible that the observed differences may have been due to minor variations in Hu protein epitopes expressed in different neuronal populations. Because information concerning clinical syndromes associated with these samples was not available, it could not be determined whether this difference in cytotoxicity for cultured cells corresponded to sites of neurological deficits in living patients.

### Intraneuronal anti-Hu immunoglobulin G accumulation and cell death require specific antibody binding to the HuD antigen

**Figure 8:**
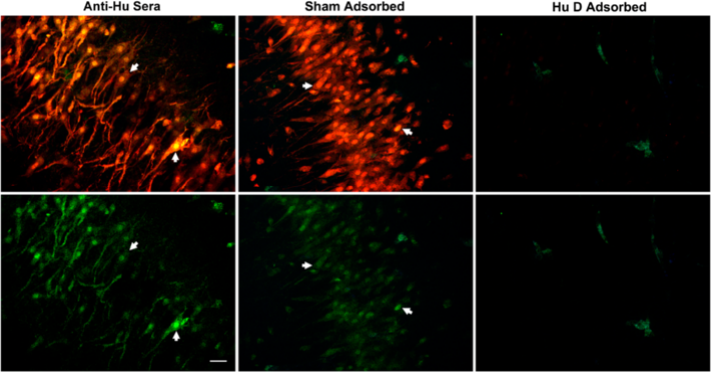
**Abolition of anti-Hu cytotoxicity for hippocampal neurons by adsorption of anti-Hu immunoglobulin G with HuD antigen.** Rat hippocampal slice cultures were incubated for 72 hours with either native anti-Hu serum (Anti-Hu Sera), the same anti-Hu antibody after passage through a nickel column with bound control vector lacking HuD protein (Sham Adsorbed), or following passage through a nickel column with bound HuD protein (Hu D adsorbed). The upper panels show merged antibody labeled with Cy5 (red) and SYTOX (green). The lower panels demonstrate neurons analyzed only for uptake of SYTOX dyes indicative of cell death. Examples of antibody-positive dead cells are shown with arrows. Adsorption of anti-Hu serum with HuD protein effectively abolished intracellular antibody binding and killing, confirming that cell death was specifically due to interaction of anti-Hu antibody with its target antigen. Similar abolition of antibody uptake and cell killing was also seen in cerebellar cultures (Additional file 3). Images are representative of studies carried out in triplicate. Magnification bar indicates 20 μm.

Because our studies involved incubation of cultures with sera containing anti-Hu antibodies, it was essential to determine the extent to which antibody binding and cell death observed in our cultures was associated with antibodies binding to Hu antigens and was not caused by antibodies to other intracellular neuronal proteins or other components which might be present in the clinical samples tested. To determine the role of anti-Hu antibodies in causing cell death, serum IgG from one patient with high titer of anti-Hu antibody response, demonstrated to produce extensive neuronal death in cultures, was adsorbed using His-tagged HuD expression protein bound to a nickel column. Serial three-fold dilutions of the adsorbed serum were then incubated with cerebellar and hippocampal slice cultures. The same serum passed through a sham nickel column, with bound control vector but without Hu antigen, was used as a control for nonspecific adsorption (see Methods). Cultures were evaluated for cell death using SYTOX and FLICA methods and were compared with amounts of cell death seen in cultures incubated with unadsorbed sera. The unadsorbed and sham-adsorbed samples produced robust neuronal death. In contrast, adsorption with Hu antigen essentially abolished intracellular accumulation of IgG, and neuronal death was reduced to background levels seen in control cultures incubated with normal human IgG (Figure 8, Additional file 3).

### Effect of anti-Ri antibodies on cerebellar and hippocampal neurons

**Figure 9:**
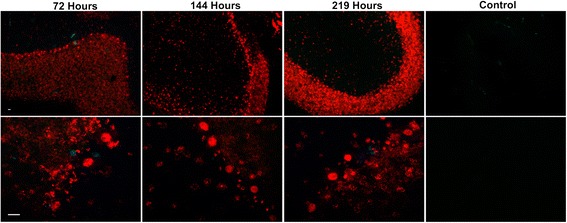
**Anti-Ri antibody did not produce neuronal death despite prolonged incubation.** Cultures of rat cerebellum were incubated with anti-Ri or control antibody and followed for 219 hours (“Control” is shown at 219 hours). Images shown demonstrate slice cultures imaged at each time point at 20× (upper panels) and 60× (lower panels) magnification. Although antibody uptake occurred in neurons throughout the cerebellum, staining by SYTOX dyes, indicating cell death, and fluorochrome-labeled inhibitors of caspases (FLICA), indicating apoptosis, did not exceed that seen in control cultures at the same time points. A similar lack of cytotoxicity over this same period was observed in hippocampal slice cultures incubated with anti-Ri antibody (see Additional file 4). Images are representative of studies employing anti-Ri sera from both patients, carried out in triplicate. Magnification bar = 20 μm.

The effect of anti-Ri antibodies on cerebellar and hippocampal cultures differed markedly from that induced by anti-Hu antibodies. Cultures incubated with anti-Ri or control antibodies were followed for up to 219 hours. Although extensive IgG uptake was observed in neurons throughout both cerebellum (Figure 9) and hippocampus (Additional file 4), cell death and apoptosis, as determined by SYTOX and FLICA methods, were not observed beyond background levels seen in controls.

## Discussion

The present study, using organotypic cultures of cerebellum and hippocampus, demonstrated that IgGs containing anti-Hu or anti-Ri were readily taken up by multiple populations of neurons and accumulated intracellularly. Demonstration of antibody uptake by neurons in real time ruled out the possibility that detection of intraneuronal IgG following incubation with either antibody could have been an artifact of fixation and processing. Detection of intracellular IgG in neurons excluding SYTOX cell death dyes confirms that antibody uptake had occurred in viable neurons. Our findings in slice cultures thus demonstrated that IgG antibodies could be taken up by living neurons and bind to their intracellular antigenic targets. Although it has been held that viable neurons exclude IgG, the ability of antibodies to enter neurons and bind to their intracellular antigens has recently been reported by Congdon and colleagues in studies employing monoclonal antibodies reactive with tau protein in a mouse model of Alzheimer’s disease [43,44]. Our observations, along with those of Congdon and colleagues, indicate that antibodies are capable of entering neurons and that antibodies could thus interact with intraneuronal antigens in other paraneoplastic or autoimmune disorders.

Although both anti-Hu and anti-Ri IgGs were incorporated into hippocampal and cerebellar neurons, the effect of these two antibodies on neuronal viability was markedly different. Anti-Hu antibody caused death of multiple neuronal populations in both cerebellar and hippocampal cultures. Neuronal death in cultures incubated with anti-Hu IgG was abolished following adsorption with the HuD antigen. These observations indicate that the cytotoxic effect of the anti-Hu sera and IgGs used in our studies was due to antibody interaction with Hu antigens and was not caused by other antibodies or factors present in patient sera. Our data indicate that anti-Hu antibodies were cytotoxic and may have a direct role in the pathogenesis of paraneoplastic neuronal injury. Although these observations do not exclude a concomitant role for T cell-mediated immunity, our study also showed that anti-Hu antibody could cause neuronal death in the absence of T cell-mediated immune response or antibody-dependent cellular cytotoxicity. The patient-to-patient variation in cell killing by individual sera containing anti-Hu antibodies may have been due in part to differences in antibody titer, and because clinical history was not available from most of the patients studied, we do not know the rapidity with which neuronal cytotoxicity might have occurred. However, this variation is also consistent with our prior observations that paraneoplastic autoantibodies strongly reactive with neurons in human brain sections may vary considerably in their reactivity with nonhuman neuronal tissue such as the rat tissue used in the present experiments [45]. This finding also parallels the differences in killing by different sera - despite similar antibody titers against frozen sections of human brain - which we have previously observed in dispersed rat cerebellar granule cell cultures [31].

Our finding that anti-Hu antibody induced neuronal cell death is in contrast to observations reported by Sillevis Smitt and colleagues [33]. These investigators were unable to produce neurological injury in mice, rats, and guinea pigs by immunization with species-specific HuD antigen or, in mice, by intravenous infusion of human anti-Hu IgG [33]. However, their study differed from ours in three important aspects. First, unlike our studies in slice cultures, their investigations were carried out in animals whose blood-brain barriers appeared to be intact, possibly limiting entry of IgG into brains and neurons. Failure of IgG to spread into brain parenchyma and interact with neurons in their study was evidenced by the absence of extravascular or intraneuronal IgG in brains when animals employed in their experiments were perfused to remove intravascular blood and antibody. Second, mice in their study were harvested no later than 48 hours after receiving intravenous human anti-Hu IgG. In the present study, slice cultures studied at 48 hours showed IgG uptake but no evidence of cell death above levels seen in controls. Third, cell death in their study was identified only by morphological methods, whereas our study used markers for both cell death and apoptosis, allowing for actual quantitation of neuronal destruction.

In previous studies, we have demonstrated that anti-Yo antibodies, associated with paraneoplastic cerebellar degeneration, accumulated intracellularly within Purkinje cells and induced non-apoptotic Purkinje cell death [34]. In the present study, uptake and cytotoxic effect of anti-Hu antibody on rat brain slice cultures differed from that of anti-Yo antibody in two aspects. First, anti-Hu antibody accumulated in and induced death of multiple neuronal populations, whereas anti-Yo antibody-associated cytotoxicity involved only Purkinje cells [34]. Second, although anti-Yo antibodies mediated non-apoptotic cell death, anti-Hu antibodies appeared to cause cell death, at least in part, by apoptosis. Our findings are consistent with those of Furneaux and Wong, who demonstrated apoptosis in BE2-N neuroblastoma cells incubated with anti-Hu antibodies [46], and by De Giorgio and colleagues, who reported production of apoptosis by anti-Hu antibodies in cultures of SH-Sy5y neuroblastoma cells and in primary cultures of guinea pig mesenteric ganglion neurons [47].

The exact mechanisms by which anti-Hu antibodies might cause neuronal death are currently unknown. Hu proteins are a group of RNA-binding proteins which share homology with the drosophila protein groups Elav and sex-lethal [48]. Four closely related Hu antigens have been cloned and sequenced: HuA (also termed HuR), HuB (Hel-N1), HuC, and HuD [48]. All of these except HuA are expressed specifically in neurons and are involved in neuronal development [48]. In older mice and rats, Hu proteins have also been shown to be important in neuronal plasticity and memory [48]. Work by Ince-Dunn and colleagues has demonstrated changes in levels of glutamate transmitter and neuronal excitability in brains of HuC^−/−^ mice (Elav13^−/−^) [49]. Although Hu proteins have been thought to affect multiple aspects of the post-transcriptional RNA function, the present study is the first to provide evidence that interaction of anti-Hu antibody with the HuD protein has a direct effect upon neuronal viability. There is extensive sequence identity between HuB, HuC, and HuD [48], and patient anti-Hu antibodies reactive with HuD have been shown to react with all four Hu antigens [50]. As such, antibodies binding to HuD could potentially bind to and interfere with the functions of other Hu antigens. The failure of anti-Ri antibodies to produce neuronal death over a more prolonged time period, despite extensive neuronal uptake, indicates that anti-Hu-mediated neuronal death is not simply a consequence of intracellular antibody accumulation.

Although we tested a smaller number of anti-Ri samples in the current study, our findings that anti-Ri antibodies did not cause neuronal cytotoxicity during the period of observation were consistent among the cultures and were in keeping with our earlier report that anti-Ri antibody did not produce death in dispersed cultures of cerebellar granule cells [51]. Our current data neither prove nor disprove a role for anti-Ri antibody in the pathogenesis of paraneoplastic neurological disease. Absence of detectable neuronal death in cultures incubated with anti-Ri antibody could possibly indicate that anti-Ri antibody is nonpathogenic. However, inability to induce cell death could also have been due to technical factors such as differences in antibody titer as compared with anti-Hu, diminished reactivity of the antibodies against nonhuman neuronal antigens as compared with those present in human neurons, or a requirement for extended exposure beyond the period of observation than that used in the present study. The major neuronal antigens recognized by anti-Ri antibodies, Nova1 (neuro-oncological ventral antigen-1) and Nova2 (neuro-oncological ventral antigen-2) represent neuron-specific RNA processing factors which control the alternative splicing of a wide array of transcripts important for synaptic activity [52,53]. Buckanovich and colleagues have demonstrated that anti-Ri antibodies interfered with the binding of the Nova-1 protein to RNA *in vitro* [54], and it is possible that the antibody could exert a similar effect in living cells.

Patients with anti-Ri antibody response differ from those with anti-Hu antibody response in that clinical improvement following immunosuppression and/or tumor removal has been reported in a number of patients [5,6,18,23–28]. Only four patients with anti-Ri antibody have been studied at autopsy, however, and only two of these had exhibited a response to treatment [3,4,23,24]. All four patients had varying degrees of neuronal loss in cerebellum, brainstem, or spinal cord, but brainstem findings were minimal in the two patients whose opsoclonus and other symptoms improved following treatment [23,24]. Our finding that the interaction of anti-Ri antibody with its target antigen was nonlethal over the time period studied thus raises the possibility that anti-Ri antibody might, at least initially, be capable of impairing neuronal function without causing cell death. This is in contrast to the irreversible clinical outcome seen in virtually all patients with anti-Hu antibody, where our current experiments suggest that neurological deficits may result from antibody-mediated neuronal destruction.

## Conclusion

Our study demonstrated that two major paraneoplastic autoantibodies, anti-Hu and anti-Ri, were both taken up by neurons throughout both hippocampus and cerebellum, and that anti-Hu antibody caused neuronal death whereas anti-Ri antibody did not affect neuronal survival. Neuronal death produced by anti-Hu antibody involved binding to the intracellular Hu antigen and occurred in the absence of T cells or Fc receptor-positive immune cells, suggesting that this antibody-antigen interaction may be directly responsible for paraneoplastic neuronal death. The lack of cytotoxic effect seen in cultures incubated with anti-Ri antibody raises questions as to whether anti-Ri antibody, unlike anti-Hu antibody, might impair neuronal function without producing cell death. The ability of antibodies to enter neurons and bind to intracellular antigens may play a key role in human paraneoplastic neurological disease and could be involved in other autoimmune or infectious central nervous system disorders.

## Additional files

Additional file 1:
**Video of contiguous serial confocal images of Purkinje and other neurons within cerebellar slice cultures incubated with anti-Hu antibody for 72 hours.** Antibody binding can be detected in both cytoplasm and nuclei of Purkinje and other neurons. Images were made at 60× and 100× magnification. Imaged cells excluded SYTOX dyes (data not shown), indicating that the antibody uptake had occurred in living cells. Similar intraneuronal distribution of IgG was also observed after incubation with anti-Ri antibody. Additional file 2:
**Uptake of anti-Hu antibodies by cerebellar neurons was followed by neuronal death, similar to that seen in studies of hippocampal neurons.** Cerebellar slice cultures from rat pups at 23 to 24 days of age were incubated for 72 hours with a 1:400 dilutions of either anti-Hu or normal sera. SYTOX green was added 2 hours prior to harvesting as a marker of cell death. Cultures were fixed and immunostained with Cy5-conjugated donkey anti-human IgG (red). Multiple Purkinje and other neurons exhibited yellow fluorescence, due to the presence of both IgG and SYTOX green, indicative of cell membrane disruption and death. Images shown are representative of experiments investigating all seven anti-Hu samples studied. IgG uptake was not observed in control cultures incubated with normal human IgG, and only rare neurons stained with SYTOX dyes. Magnification bar = 20 μm. Additional file 3:
**Abolition of anti-Hu cytotoxicity for cerebellar neurons by adsorption of anti-Hu IgG with HuD antigen.** Rat cerebellar slice cultures were incubated for 72 hours with native anti-Hu serum (Anti-Hu serum), the same anti-Hu antibody after passage through a nickel column with bound vector lacking HuD protein (“Sham-absorbed”), and following passage through a nickel column with bound HuD protein (HuD adsorbed). The upper row of figures shows merged antibody labeled with Cy5 (red) and SYTOX (green). The lower row of figures shows SYTOX staining only, indicative of cell death. Examples of antibody-positive dead cells are shown with arrows. As in studies of hippocampal cultures, adsorption of anti-Hu serum with HuD protein effectively abolished intracellular antibody binding and killing of cerebellar neurons (Figure 6), again confirming that cell death was specifically due to interaction of anti-Hu antibody with its target antigen. Magnification bar = 20 μm. Additional file 4:
**Antibody uptake but lack of cytotoxicity in cultures of rat hippocampus incubated with anti-Ri antibody.** Slice cultures of rat hippocampus were incubated with anti-Ri antibody and followed for 219 hours. Although antibody uptake was observed in neurons throughout the hippocampus, staining by SYTOX dyes, indicating cell death, or by FLICA, indicating apoptosis, did not exceed that seen in control cultures at the same time points. Magnification bar = 20 μm.

## Abbreviations

FLICA: fluorochrome-labeled inhibitors of caspases
Ig: immunoglobulin
TUNEL: terminal deoxynucleotidyl transferase dUTP nick end labeling
