# Supplementary figures and images for: Neuronal uptake of anti-Hu antibody, but not anti-Ri antibody, leads to cell death in brain slice cultures
Source: J Neuroinflammation. 2014 Sep 17;11:160. doi: 10.1186/s12974-014-0160-0 (PMC4174281; doi:10.1186/s12974-014-0160-0)

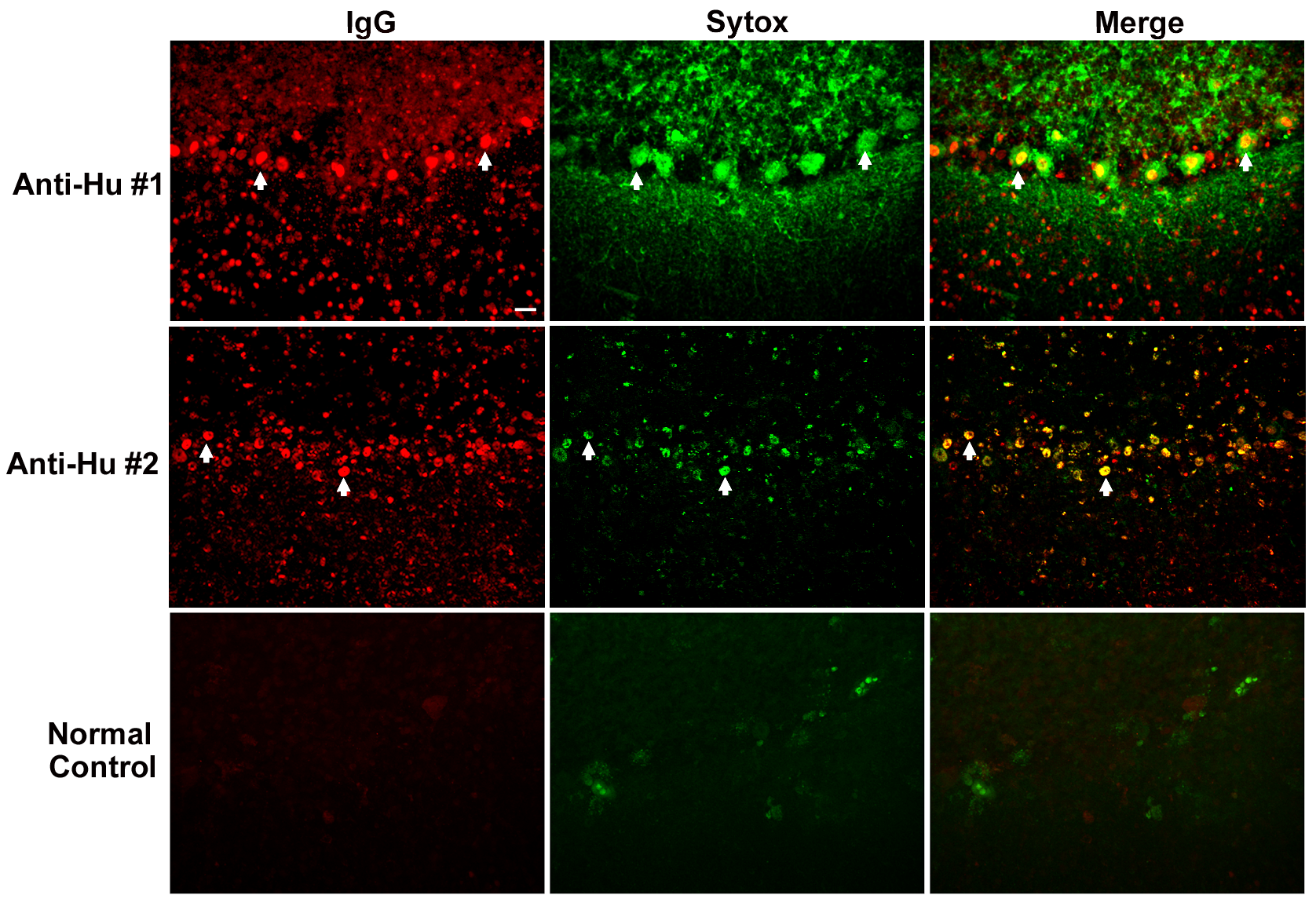

Supplement: Additional file 2: — Uptake of anti-Hu antibodies by cerebellar neurons was followed by neuronal death, similar to that seen in studies of hippocampal neurons. Cerebellar slice cultures from rat pups at 23 to 24 days of age were incubated for 72 hours with a 1:400 dilutions of either anti-Hu or normal sera. SYTOX green was added 2 hours prior to harvesting as a marker of cell death. Cultures were fixed and immunostained with Cy5-conjugated donkey anti-human IgG (red). Multiple Purkinje and other neurons exhibited yellow fluorescence, due to the presence of both IgG and SYTOX green, indicative of cell membrane disruption and death. Images shown are representative of experiments investigating all seven anti-Hu samples studied. IgG uptake was not observed in control cultures incubated with normal human IgG, and only rare neurons stained with SYTOX dyes. Magnification bar = 20 μm. [file 12974_2014_160_MOESM2_ESM.tiff]

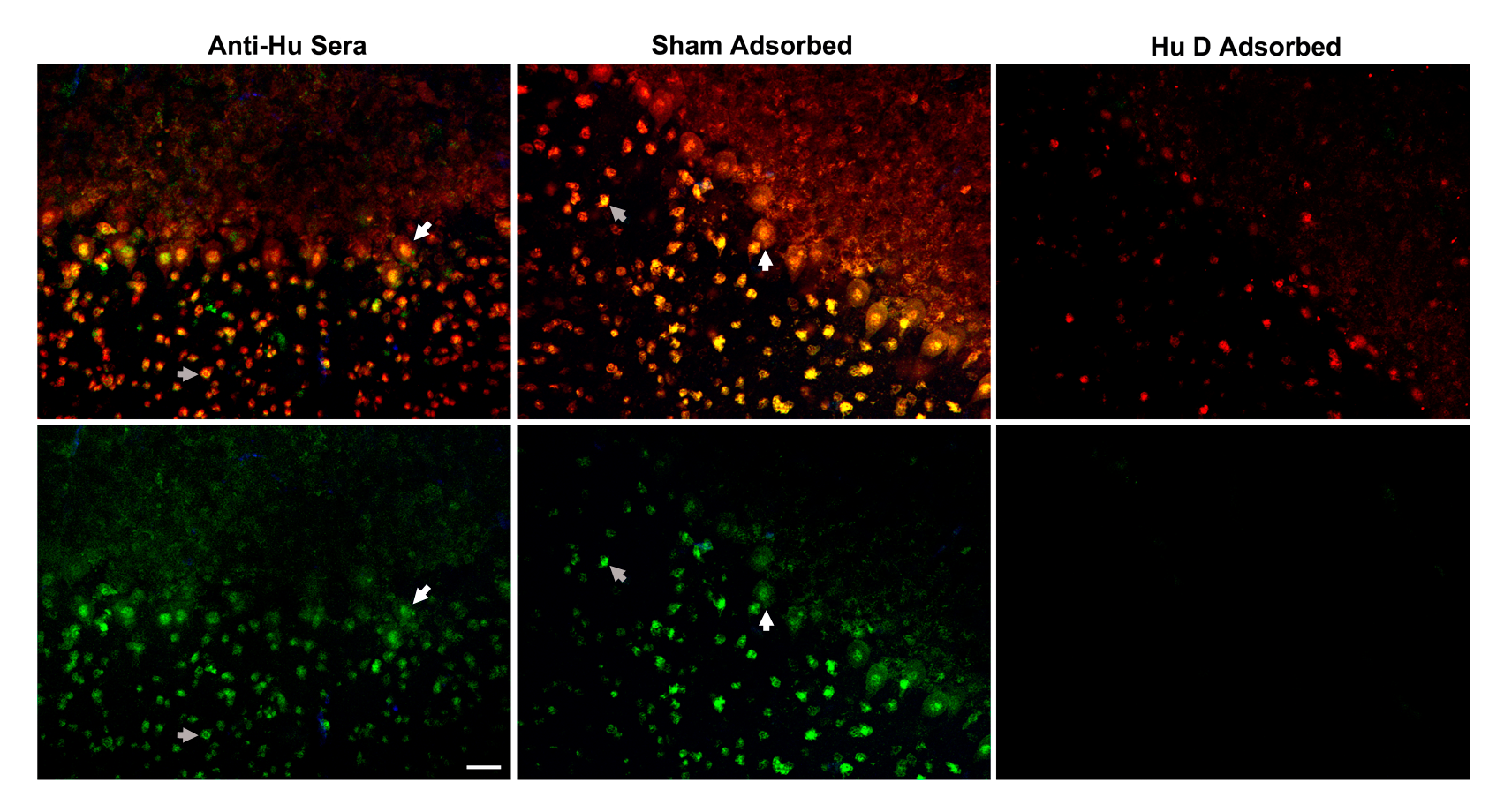

Supplement: Additional file 3: — Abolition of anti-Hu cytotoxicity for cerebellar neurons by adsorption of anti-Hu IgG with HuD antigen. Rat cerebellar slice cultures were incubated for 72 hours with native anti-Hu serum (Anti-Hu serum), the same anti-Hu antibody after passage through a nickel column with bound vector lacking HuD protein (“Sham-absorbed”), and following passage through a nickel column with bound HuD protein (HuD adsorbed). The upper row of figures shows merged antibody labeled with Cy5 (red) and SYTOX (green). The lower row of figures shows SYTOX staining only, indicative of cell death. Examples of antibody-positive dead cells are shown with arrows. As in studies of hippocampal cultures, adsorption of anti-Hu serum with HuD protein effectively abolished intracellular antibody binding and killing of cerebellar neurons (Figure 6), again confirming that cell death was specifically due to interaction of anti-Hu antibody with its target antigen. Magnification bar = 20 μm. [file 12974_2014_160_MOESM3_ESM.tiff]

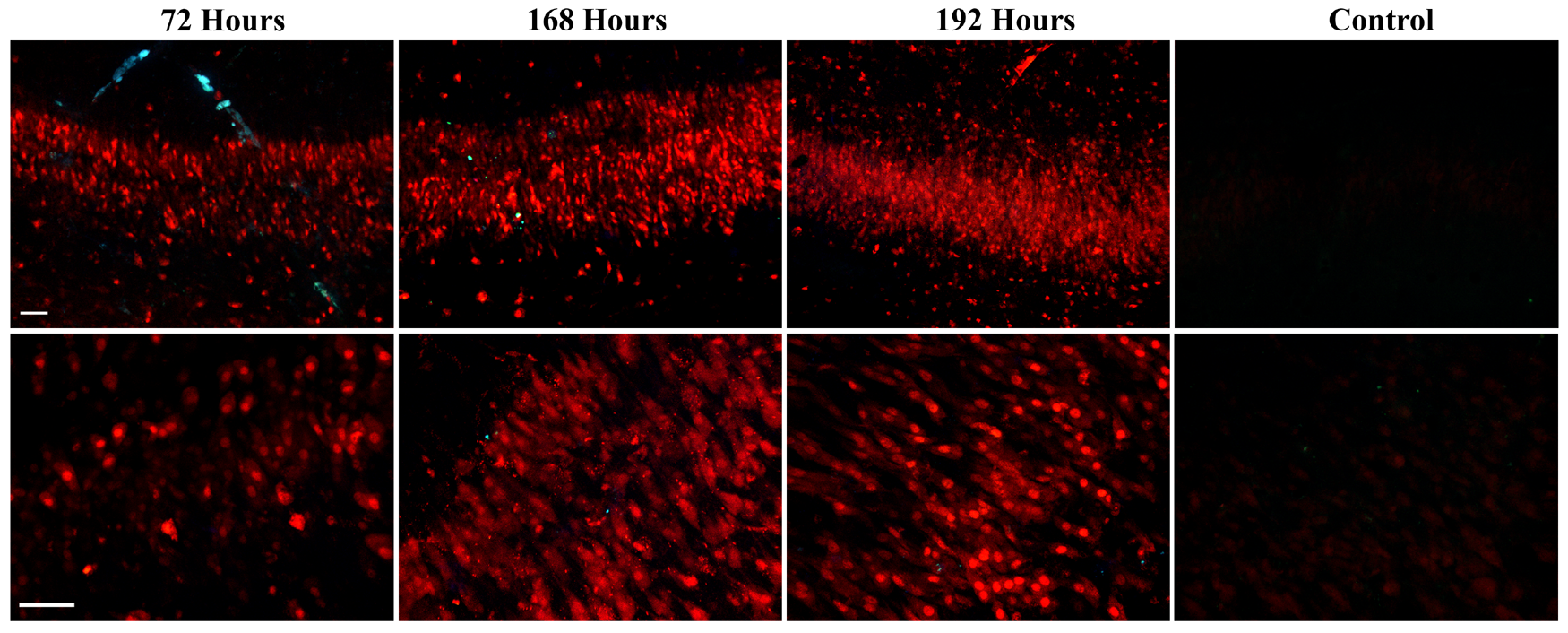

Supplement: Additional file 4: — Antibody uptake but lack of cytotoxicity in cultures of rat hippocampus incubated with anti-Ri antibody. Slice cultures of rat hippocampus were incubated with anti-Ri antibody and followed for 219 hours. Although antibody uptake was observed in neurons throughout the hippocampus, staining by SYTOX dyes, indicating cell death, or by FLICA, indicating apoptosis, did not exceed that seen in control cultures at the same time points. Magnification bar = 20 μm. [file 12974_2014_160_MOESM4_ESM.tiff]
